# A qualitative comparison of experiences of specialist mother and baby units versus general psychiatric wards

**DOI:** 10.1186/s12888-019-2389-8

**Published:** 2019-12-16

**Authors:** Jessica Griffiths, Billie Lever Taylor, Nicola Morant, Debra Bick, Louise M. Howard, Gertrude Seneviratne, Sonia Johnson

**Affiliations:** 1 Division of Psychiatry, University College London, Maple House, 149 Tottenham Court Road, London, W1T 7NF UK; 2 Warwick Clinical Trials Unit, Warwick Medical School, University of Warwick, Gibbet Hill, Coventry, CV4 7AL UK; 3 Section of Women’s Mental Health, Institute of Psychiatry Psychology and Neuroscience, King’s College London, Crespigny Park, London, SE5 8AF UK; 4 South London and Maudsley NHS Foundation Trust Channi Kumar Mother and Baby Unit, Bethlem Royal Hospital, Monks Orchard Road, Beckenham, BR3 3BX UK

**Keywords:** Perinatal, Mental health, Women, Mother and baby unit, Acute psychiatric ward, Qualitative

## Abstract

**Background:**

Mother and baby units (MBUs) are an inpatient mental health service where women experiencing acute severe postpartum psychiatric difficulties can be admitted with their babies. They are currently viewed as best practice in the UK and elsewhere. However, as service provision is fragmented, some women residing in areas without MBUs are admitted to acute general psychiatric wards without their infants. This study aimed to compare qualitatively experiences of these two service types from the perspectives of women and clinicians.

**Methods:**

Semi-structured interviews were conducted with fifteen women who received treatment for perinatal mental health problems on a general psychiatric ward and/or MBU in England. Two focus groups were also conducted, one with MBU staff (*n* = 11) and one with acute ward staff (*n* = 6). Data were analysed thematically.

**Results:**

Women generally preferred being co-admitted with their baby to an MBU over lone admission to a general psychiatric ward. Women and clinicians felt that MBUs provided more perinatally-focused, family-centred care, and were better-equipped to meet women’s needs. General wards were reported by women and staff to lack the necessary facilities and expertise to support perinatal women adequately, while separation of mothers and babies was often experienced by women as traumatic and detrimental to recovery. However, some areas for improvement were also identified across both service types, particularly relating to difficulties transitioning home post-discharge, inadequate support for family members, staffing issues and access problems (with MBUs).

**Conclusions:**

Findings suggest that specialist perinatal inpatient care is considered preferable to generic care in the perinatal period from both service user and staff perspectives. Increased collaboration between perinatal and non-perinatal services could help improve perinatal expertise on general psychiatric wards, while further expansion of perinatal services (e.g. to cater for women currently considered too high risk for MBUs and for those discharged from inpatient settings) could tackle other shortfalls in care.

## Background

As many as one in five women will develop some form of mental health problem during the perinatal period, usually defined as pregnancy and the year following birth [1]. There is also an increased risk of first onset or recurrence of severe mental health difficulties at this time, with 1–2 per 1000 women admitted to hospital for treatment after giving birth [2, 3]. The consequences of perinatal mental health difficulties can be severe, wide-ranging and costly [4, 5] but timely access to appropriate treatment can reduce the risk of adverse outcomes [6].

It has been argued, both in the UK and internationally, that perinatal women require care that is more specialised than is provided by general adult mental health services [7, 8]. Mother and baby units (MBUs) are a specialist model of inpatient care for women experiencing severe perinatal psychiatric difficulties. They enable women to be co-admitted full-time with their babies, rather than being separated from them, as they would be if admitted to a general psychiatric ward. Whereas staff on general psychiatric wards do not necessarily have any specialist perinatal expertise, multidisciplinary teams on MBUs are trained in the treatment of perinatal mental health problems, and in childcare and development [9]. MBUs predominantly admit women with psychotic disorders, mania, and severe depression but also support those with severe forms of other conditions, such as obsessive-compulsive disorder or anxiety.

Research exploring satisfaction with MBUs has shown that women’s experiences are generally positive. In particular, they value infant-care advice on MBUs, readily available baby equipment, good visitor arrangements for partners/relatives, the comfort of the environment, and being able to remain with their babies [10, 11]. However, although MBUs are viewed as best practice in some countries (particularly in the UK, France and Australia) [12], and exist in a number of countries worldwide (including the US, India, Israel and Sri Lanka), access is often inequitable due to their highly disparate geographical distribution and small overall numbers of beds [9]. In many countries, there is no MBU provision. In the UK, the planned opening of four new units will mean there are twenty-one MBUs across the UK by 2020/21 [8]. However, although this is more than in most countries, it is still insufficient to cater for the level of demand, meaning some women who need in-patient admission will be admitted to a non-specialist general psychiatric ward. It is therefore important to understand women’s experiences of both service contexts to inform future research, policy-making and mental health service commissioning, both in the UK and internationally.

Much research has explored service users’ experiences of hospital-based acute psychiatric care generally. Service users widely express dissatisfaction, often experiencing general wards as non-therapeutic, restrictive and frightening environments, lacking access to privacy and holistic care, and inadequately involving them in decisions [13–15]. Poor relationships with staff are also commonly reported, due to factors such as low staffing levels and a lack of staff continuity [15–19].

However, few studies have explored perinatal women’s experiences of general psychiatric wards or directly compared these with MBUs. Two small qualitative studies, in Canada and England, found that women experiencing postpartum psychosis preferred MBUs to general psychiatric wards [20, 21]. Women experienced frustration at the lack of specialised perinatal support on general wards and struggled to cope with the separation from their babies. However, women’s views of services were only explored very briefly in these studies, as part of a wider remit, so the conclusions were limited. In Scotland, a wider-ranging mixed-method study similarly found that women preferred MBUs to general wards, and viewed remaining with their babies as one of the most important aspects of their care [22]. This study found that MBUs offered greater perinatal expertise, better peer support, a stronger recovery focus, and greater service user and family involvement than general psychiatric wards. The findings also raised concerns about the support provided on general psychiatric wards for perinatal women, in particular: lack of consideration of children in patients’ records, care plans and risk assessments; lack of supported contact between mother and baby; and inadequate provisions for visiting families.

The current study builds on this previous research, exploring in-depth how MBUs are experienced in comparison to general psychiatric wards, and identifying potential areas for service improvement. This has the potential to inform the development of services both nationally and internationally. Women’s views were complemented by the views of MBU and acute ward clinicians, so as to understand staff perspectives too and identify possible solutions to issues raised by women.

## Methods

This study was part of a wider qualitative research study (known as the STACEY study), exploring experiences of a range of services treating women with perinatal mental health difficulties. The STACEY study was itself part of a wider mixed-methods programme of research called Effectiveness of Services for Mothers with Mental Illness (ESMI) [https://www.kcl.ac.uk/ioppn/depts/hspr/research/ceph/wmh/projects/a-z/esmi]. The current study takes a qualitative descriptive approach, with a critical realist orientation [23, 24].

### Participants

#### Women

Fifteen women from the wider STACEY sample (of 52 women) were included in this study because they had been admitted to a general psychiatric ward and/or specialist MBU for an acute severe psychiatric problem during or soon after their most recent pregnancy. These fifteen women were recruited from nine NHS healthcare providers across England. For the wider STACEY study, purposive sampling was used to ensure women with a range of clinical and demographic backgrounds were included. Additional inclusion criteria required that women: were aged 16 or over; English language speakers; and had a baby aged 6–9 months at the time of interview. Individuals lacking capacity to consent were excluded. Eligible women were initially identified and approached by a clinician from their mental health team. Those who expressed an interest in participating were then contacted by a researcher to provide more information about the study and interview those who were willing.

#### Clinicians

Seventeen clinicians were recruited to two focus groups (the wider STACEY study included 10 focus groups in total with clinicians from a range of different services). One of the two focus groups consisted of clinicians working on an MBU (*n* = 11), the other consisted of clinicians working on a female acute psychiatric ward (*n* = 6). Clinicians were recruited to focus groups by contacting the relevant services and, with their support, advertising the study within these services. Inclusion criteria required that clinicians had experience of supporting women with perinatal mental health problems.

NHS ethical approval was obtained (reference: 13/LO/1955) and all participants provided informed written consent.

### Data collection

#### Women

A semi-structured interview schedule was developed by the research team and was reviewed by a perinatal service user and carer advisory group (PAG). As the wider STACEY study explored women’s experiences of their entire care pathway, interviews explored women’s views of all the services they accessed for their perinatal mental health. In cases where interviews covered views of other services as well as MBUs/acute wards, only the relevant MBU/acute ward data was analysed for this study. The interview schedule was piloted with five women. It was first piloted with a woman who had stayed on an MBU and was part of the PAG. The interview guide was subsequently adapted and then piloted with four other women, recruited from NHS services, who were eligible for the STACEY study. This included one woman who had stayed on an MBU, who was included in the current study since she met eligibility requirements and the interview schedule required very little changing after this stage of piloting. Individual semi-structured interviews were conducted with participants each lasting approximately one hour, usually at the participant’s home.

#### Clinicians

Preliminary anonymised findings from interviews with women were presented to clinicians in focus groups. Clinicians were asked to discuss these findings in relation to their own experiences of supporting women with perinatal mental health difficulties. Focus groups lasted approximately one hour.

Both focus groups and most interviews (*n* = 13) were conducted by the second author (a clinical psychologist and researcher). Two interviews were carried out by a postgraduate student and by a professor of social work. In some cases, researchers involved in the study were also working clinically in areas from which we recruited. The professor assisted in conducting interviews in locations less accessible to the main researcher, since the study covered a wide geographical area. The postgraduate student was involved in conducting interviews to gain experience as they were doing research in perinatal mental health. Having multiple interviewers introduced diversity in interviewing styles, which may have helped to elicit more varied responses from participants, especially since interviewers had different ethnic backgrounds. Actions taken to help ensure consistency included using a semi-structured interview guide, and the fact that the postgraduate student was supervised and accompanied by the main researcher.

### Analysis

Interviews/focus groups were audio-recorded, transcribed verbatim, and anonymised. Data were analysed by the first author using thematic analysis [25] with NVivo software. Commonalities and variations in the data were explored, focussing on how these related to the two different service contexts being investigated, through an iterative, staged approach utilising inductive and deductive techniques. Themes were refined and reorganised throughout the analysis process, resulting in a final coding frame.

In qualitative research, reflexivity is seen as vital, with researchers encouraged to acknowledge the ways in which their own backgrounds, beliefs and positioning inevitably affect the research [26]. In this study, for example, the main interviewer was a White British clinical psychologist (and mother) with experience of working within perinatal mental health services. Such factors could have influenced her assumptions and approach to the study, as well as participants’ responses to her. The research team was also all-female, and the lack of a male perspective could have influenced data collection, analysis and the conclusions drawn.

To enhance rigour, a collaborative approach to coding was adopted with a second researcher producing a separate coding frame, based on a sub-sample of the transcripts. The two coding frames were compared and discussed, with competing explanations of the data explored. The initial coding frame was then revised in line with insights gained from this discussion.

## Results

### Participant characteristics

Fifteen women were recruited. They described accessing five different MBUs and thirteen general psychiatric wards during and/or after their most recent pregnancies. Six had accessed general psychiatric wards only, six had accessed MBUs only, and three had experience of both service contexts. A summary of their characteristics is shown in Table 1. Participants ranged from 19 to 39 years old. Most were from a white British or white other background and lived with their partner.

**Table 1:** Characteristics of women (*N* = 15)

Characteristics	Category	Respondents n
Service use	MBU only	6
	General ward only	6
	Both	3
Age	Mean age	32 years
	< 20 years	1
	20–29 years	4
	30–39	10
Ethnicity	White British	8
	White Other	2
	Black Caribbean	2
	Black African	1
	Asian	1
	Mixed Race	1
Primary diagnosis (self-reported)	Depression	4
	Bipolar disorder	2
	Postpartum psychosis	5
	Schizophrenia	1
	Personality disorder	3
Previous mental health service use	Yes	11
	No	4
Level of education	No formal qualifications	1
	Secondary education	7
	Undergraduate	1
	Postgraduate	6
Living with partner	Yes	12
	No	3
Custody status	Retained custody of baby	13
	Lost custody of baby	2
Number of children	1	9
	2	6

Clinician characteristics are shown in Table 2. Clinicians varied in their clinical roles, and those in the acute ward group were more diverse by ethnicity and gender than the MBU group.

**Table 2:** Characteristics of clinicians (*N* = 17)

Characteristics	Category	Respondents n
Mother and baby unit (MBU) focus group (*n* = 11)		
Gender	Female	10
	Male	1
Ethnicity	White British	9
	White Other	1
	Black African	1
Role	Mental health nurse/student nurse	5
	Nursery nurse	2
	Healthcare assistant	1
	Psychologist	1
	Occupational therapist	1
	Senior manager	1
General ward focus group (*n* = 6)		
Gender	Female	3
	Male	3
Ethnicity	White British	1
	White Irish	1
	White Other	1
	Black African	2
	Black Caribbean	1
Role	Foundation doctor	2
	Modern matron	1
	Ward manager	2
	Student nurse	1

### Findings

Women expressed a clear preference for co-admission with their baby to an MBU over lone admission to a general psychiatric ward. Women and clinicians alike described acute wards as struggling to care adequately for perinatal women in the face of a lack of appropriate facilities and expertise. In contrast, MBUs were generally perceived by women and clinicians to be better-equipped to provide family-inclusive, perinatally-focused care, within more family-friendly and therapeutic environments. Women with experience of both settings invariably preferred MBUs. However, both women and clinicians also identified areas for improvement on MBUs, such as increased accessibility and improved post-discharge continuity of care. Key themes and sub-themes are outlined in Table 3.

**Table 3:** Summary of the themes and sub-themes identified in this study

Themes	Sub-themes
Degree of perinatal focus in care	Expert, tailored support
	Co-admission versus separation of mother and baby
Family involvement	Family contact/visits
	Family inclusion/support
Therapeutic relationships and environment	Peer support
	Therapeutic relationships with staff
	Therapeutic environment
Access and post-discharge experiences	Barriers to access
	Poor continuity of care post-discharge

#### Degree of perinatal focus in care

##### Expert, tailored support

Women preferred the more specialist, perinatally-focussed care provided by MBUs to the more generic support on general psychiatric wards. Tailored support included: activities designed for new mothers and their babies (some of which could be attended by both mother and baby), breastfeeding support, parenting interventions, help with infant-care, specialist advice around medication, and mother-baby relationship interventions. Those on MBUs described feeling they were ‘in good hands’, with a dedicated, skilled team available to help them. Some women valued clinicians’ detailed knowledge of the risks/benefits of different medications in the perinatal context. Others said perinatally-focused interventions (such as videoing interactions between mother and baby and playing them back) assisted them in developing their parenting skills and promoted a positive mother-baby attachment. Mothers also valued practical support with caring for their babies provided by nursery nurses.

*“[The psychologist] told me useful things about … how babies you know develop and different ways to hold him, or play with him or talk to him … It was useful to see the video back and see how you are acting …*

*They had so many different ways that they were trying to help, different facilities and different medical staff doing all different things, and it was very structured. And they had a lot of experience.” Mother 5*

A few women felt that, even on MBUs, support with their babies could be improved further. For example, some women found MBUs were not as well set up for older babies (e.g. where restricted access to the kitchen/cutlery made weaning difficult). Others wanted more tangible advice about aspects of infant-care (e.g. how to settle their babies or transition from breastfeeding to bottle-feeding), while some also felt they received conflicting parenting advice from different staff. However, overall women valued the mother-baby-oriented focus of MBUs, which also allowed them space to recuperate.

*“I felt, maybe, they didn’t, kind of, had quite the training in, kind of, trying to get the baby to sleep, and trying to get them into a routine, or trying to transition to bottle feed. But they were really very, they were very nice in terms of helping, offering to help with, like, I could have a shower and leave [my baby] with them, which I hadn’t been able to do, really, at home.” Mother 10*

In contrast, care on general psychiatric wards was more generic, typically lacking any perinatal focus at all. In some cases, staff were even unaware that participants had recently had a baby. This created a perception among some women that general wards were essentially trying to control or ‘manage’ them (e.g. through medication) rather than helping them more therapeutically or holistically with the wider context of motherhood.

*“The acute units, they don’t really look at the whole person, they don’t kind of care that you’ve just had a baby. It’s just, they just look at the immediate presentation that they’ve got in front of them. This person’s being difficult, what drugs can you give them. That’s very much a drugs culture …*

*[Some staff] had no idea that I was a mother separated from a new born - or very young - baby.” Mother 8*

This is not to say, however, that women could not benefit at all from this less-tailored care. Some found more generic interventions, such as medication and group therapy, therapeutically beneficial. Similarly, whilst some found activities (such as cooking, gardening, pottery, creative writing and exercise classes) irrelevant, others valued the opportunities for social interaction and structure they provided. Women were, however, usually satisfied with the variety of activities and interventions on MBUs, and usually dissatisfied with this on general psychiatric wards.

MBU and acute ward clinicians agreed that support on MBUs is typically more holistic, person-centred and perinatally-focused. They attributed problems with this on general wards to factors such as the high acuity of the environment (and consequent demands on staff) as well as limited availability of perinatal resources and expertise.

*“You’ve got all these admissions coming in, everything is moving at such a fast pace … I think we forget sometimes as a group that these are individuals who are probably going through a break period, being separated from their baby, because sometimes everything is going on.” (Acute ward, ward manager)*

General ward clinicians reported receiving little perinatal training and sometimes felt out of their depth when asked for specific advice (e.g. about breastfeeding or medication).

*“If I hadn’t randomly spent those two days on the MBU ward, we wouldn’t have received a single minute of perinatal training in our psychiatry locations and teachings.” (Acute ward, foundation doctor)*

*“Personally, just from a nursing perspective, I’ve got zero training … If you were lucky, you would end up in placement in perinatal. But I think maybe someone talked to me about it for an hour during the early years of my career.” (Acute ward, modern matron)*

Clinicians suggested that the disparity in perinatal expertise between general ward and MBU staff could be addressed by more collaborative working between services. For example, training and consultation by MBU staff or community perinatal specialists to help equip general ward staff with skills and knowledge to meet the needs of new mothers (whether this be advising on specific cases, or on wider service-level approaches and developments).

*“We’ve got a really good perinatal [community] service here and actually getting those really experienced staff to come talk to us about how you have those conversations with [women] and the family and how you help support them [would be valuable].” (Acute ward, modern matron)*

General ward staff said their ward had also been designated their hospital’s perinatal ward. They felt this had helped develop at least some perinatal expertise within the ward, in the context of limited resources. Staff from both services emphasised that improving perinatal care provision through training and development requires support from all levels of the organisation (right through to senior management).

##### Co-admission versus separation of mother and baby

Most women expressed a strong preference for co-admission with their babies over separation. Many mothers perceived sustained mother-baby contact to be an important facilitator of recovery. Separation following admission to general psychiatric wards was often viewed as distressing, a barrier to recovery, and detrimental to the mother-baby relationship, because it was felt to deprive mothers of the opportunity to bond with their baby and develop their parenting abilities. Women on general wards sometimes felt they had ‘missed out’ on being a mother and some struggled to adjust back into their mothering role after discharge.

*“It was much nicer to say to a mum, I think, that we’re going to help you to look after your child, not get somebody else to look after your child and help you. … I found it incredibly hard this year to be away from them. It was like every day I missed them.” Mother 4*

Admission to acute wards could also place a burden upon relatives to take on additional childcare responsibilities and there was one instance where a woman’s baby was taken into custody by social services because it was not feasible for her relatives to provide childcare.

Separation of women from their babies could also cause difficulties related to breastfeeding, with mothers reporting that acute wards were not well set up to support them with this.

*“My baby was exclusively breastfed before all this hospitalisation, so I was still expressing milk whilst at the hospital. They didn't have any freezers, so there was no way of my freezing the milk and getting it taken to her, so I had, I ended up having to express milk and down it through the sink.” Mother 13*

Nevertheless, a few women admitted to general psychiatric wards felt separation had been for the best because they worried they would have been unable to care for their baby themselves and had concerns about exposing them to an inpatient environment. Some felt that separation provided respite and motivation to recover.

*“It was hard but I would say that it’s good to have the mother go and recuperate somewhere else without the kids … You need that time to yourself to recuperate … So ending up at the hospital was a good thing.” Mother 12*

Co-admission also separated the baby from the rest of the family, including the father. One woman found sharing childcare responsibilities with her partner throughout her admission helped them manage this.

Clinicians echoed women’s views – especially acute ward staff, who recognised that women often experienced separation from their baby as traumatic and highly distressing. They felt that relatively simple actions, such as acknowledging and validating the women’s distress could be helpful in supporting them through this.

*“There’s simple things as well … Just asking in the ward … How are you doing today?...This must be really difficult. … You’re a new mother and then there’s no new baby with you and that must be so tough.” (Acute ward, foundation doctor)*

General ward clinicians acknowledged problems caused by a lack of facilities to meet the needs of mothers separated from their babies, especially relating to breastfeeding.

*“Another problem is when [the] patient really wants to breastfeed … it’s quite difficult. There could be a problem of breast engorgement. And all those endless limitations to what staff could be really do in that aspect. So, some of them eventually they are forced to stop breastfeeding … not able to really find an appropriate fridge to store the milk and all that stuff.” (Acute ward, ward manager)*

Staff commented that in-person visits to MBUs could help generate inspiration for possible adaptations to the ward environment to improve perinatal care provision, including simple changes. Most had never set foot on an MBU.

*“I’d really like to go and look at an MBU to understand what some things really look like. And what facilities they have. Because there might just be simple, simple stuff, like a new freezer where they can keep breastmilk in, just like really tiny things, really quick things that we could do.” (Acute ward, modern matron)*

#### Family involvement

##### Family contact/visits

Women found separation from older children, partners and other relatives during admissions challenging in both service contexts. Maintaining contact between women and their families throughout admissions was therefore considered important.

While one woman felt poor internet and phone connections made contact with her partner difficult during her stay, MBUs were typically better at facilitating family contact than general psychiatric wards, more commonly providing private family rooms and extending visiting hours to accommodate families’ schedules and needs. This was especially helpful in cases where home-MBU distances were long.

*“They noticed how far away I lived and they were like you know they can come before visiting times and stay a little after, because they lived so far and, yes, so that was really good.” Mother 1*

Fewer of these provisions on general psychiatric wards made it more difficult to sustain regular contact between women and their families.

*“That was quite frustrating as well because there is visiting times and they were very specific about it … But some people can’t make those times … There wasn’t too much flexibility and it would’ve been nice especially in that environment.” Mother 4*

In some cases, babies and children were not permitted to visit general psychiatric wards at all. However, even when they were, scheduling visits around babies’ routines could be a challenge, while some women had concerns that other patients’ behaviour could be disturbing and difficult to understand for children and so were reluctant to let them visit. Some women who stayed on general wards said there was no private family room available, making it feel like an inappropriate and unsafe environment for children.

Clinicians from both service contexts recognised that facilitating contact between women, babies, fathers and other family members is important (including for any older children) and consensus amongst clinicians was that MBUs are better set-up for this. One member of MBU staff recalled how, in the past, women’s partners had even been permitted to stay overnight on some MBUs. However, she said this had since been disallowed, due to a perception that women had a competing need for privacy. Acute ward clinicians described challenges with family visits, for example, saying that their family room was located off the ward so mother-baby contact was not permitted if the mother was not deemed well enough to leave the ward.

##### Family inclusion/support

Women commented on how difficult their partners and families found the perinatal period as well, especially in the context of a woman experiencing severe mental health difficulties. Psychological support for partners/relatives, such as individual counselling, was more commonly available on MBUs than general psychiatric wards. This family support was positively received by those who were able to access it.

*“When I went into the mother and baby [unit] … we had support as a family. All of us … They worked with us as a family as opposed to an individual.” Mother 4*

Most participants, however, reported a lack of psychological, childcare or financial support for family members in both service contexts. Even in cases where it was available, it was difficult to access due to clashes with relatives’ work and childcare commitments. In particular, long home-MBU distances, because of a limited number of MBUs unevenly distributed across the country, made travel costly and time-consuming for families.

*“The one psychologist at the MBU suggested that my partner comes along to one of the sessions. But he just couldn’t find time for it, basically, and to do his work. And also, where hospital is, it’s miles from where he works and stuff like that, it’s a few hours travelling.” Mother 3*

While there were exceptions, participants did nevertheless generally feel that family members were adequately involved in decisions on MBUs. Efforts made by MBUs to facilitate this included: communicating effectively with partners/family members to keep them well-informed, regularly inviting them to ward rounds, and actually taking their views into account. This was typically valued by women, who sometimes felt that they were too unwell to make decisions for themselves. General psychiatric wards were more often, though not always, described as failing to include family members.

Clinicians recognised that offering support to partners/family members and involving them in women’s care can be beneficial for all involved. They felt that MBUs typically offer more opportunities for family members to be supported and involved, though MBU staff felt a more systematic approach to this is still required. Indeed, clinicians acknowledged that there was room for improvement in both service contexts.

*“I think what we probably do try to do is to try and have a meeting with the partner, maybe a psycho-educational session or something to talk about what will be helpful when they go. I’m not saying that every partner gets that...I don’t know how systematically we do it. But we do recognise that’s really key actually, because I think partners don’t quite know what to say or what to do or what would be helpful, and they need some guidance.” (MBU, psychologist)*

Practical barriers such as limited time and resources due to the high demands already placed on staff could make it difficult for clinicians to achieve their goals of increased family-working.

*“I think the team find it really challenging because you can imagine … with so many patients … it’s really difficult to juggle … It’s not an easy thing to do to involve them all the time. But we aspire to do that wherever possible.” (Acute ward, modern matron)*

#### Therapeutic relationships and environment

##### Peer support

Many women valued being admitted to an MBU alongside other women experiencing perinatal mental health difficulties. Some participants felt that it made it easier for them to relate to, bond with and support each other. This helped create a therapeutic sense of ‘community’ in MBUs. Plus, seeing other women’s difficulties and mother-baby relationships improve helped to instil hope in participants. This demonstrates how peer support could facilitate recovery. In some cases, friendships were long-lasting, providing mothers with continued social support in the community.

*“Just knowing that there were other mums, it was just like the biggest comfort ever. I just felt like, oh my gosh I’m not the only one.” Mother 1*

Some participants did recall bonding with other women with older children, and other patients, on general psychiatric wards. However, this tended to be perceived as more difficult because service users had less in common and more severe and variable conditions.

*“[The ward] was a very confusing place to be and having all those people around me that didn’t understand what had happened to me was really hard.” Mother 14*

##### Therapeutic relationships with staff

As well as peer relationships, women experienced positive therapeutic relationships with staff across both service contexts. MBU staff in particular were described as working well together as a team, almost like a family. Women valued staff who were dedicated to their jobs, made them feel listened to, understood and respected, and were warm and patient. Such compassionate, non-judgemental support helped establish trust between staff and service users, especially important since many mothers reported being initially distrustful of staff.

*“I had a good relationship with the staff [on the acute ward]. They were really nice, they were courteous. And they were good listeners as well. You know, if you had a problem you could talk to them and they would try to help you out.” Mother 12*

However, women in both service contexts described some members of staff as cold, unempathetic and not proactive enough. Women attributed this to staff being too busy or lacking commitment to their jobs. It resulted in women feeling apprehensive, frustrated and less able to approach staff.

*“You could definitely see the ones [on the MBU] that were, you know, a bit more under stress and a bit more snappy. And there were the ones that actually enjoyed being there and liked caring for the babies and liked being around the mums and you could see the ones that were there just for a job sake.” Mother 1*

In both general psychiatric wards and MBUs, staff unavailability (due to understaffing or staff being too busy) was a commonly-identified barrier to therapeutic relationships with staff. This was made worse by poor staff continuity resulting from the employment of bank and agency staff to compensate for staffing shortages, since these individuals were less familiar with mothers. Staff shortages could also result in activities and visits being cancelled across both settings and staff having less time for women, compromising their quality of care.

“*I really didn’t like, you know, the fact that there is a different person looking after you every day [on the MBU], twice a day, actually, it changes … I never knew who was supposed to look after me … Plus the, what do you call the people from the agency?... Nobody bothered to tell [me] what they were supposed to do.” Mother 3*

Like women, clinicians across both service contexts identified understaffing and lack of regular permanent staff familiar with the service and its users as barriers to providing safe, quality care. Plus, the high demands placed on staff could result in more process-focussed, and less person-centred care; in both service contexts, clinicians said there was a lack of time for them to cater to women’s unique individual needs.

*“We don’t have enough staff to meet our safe staffing levels which is something that they’re about to fix I hope. But we can’t actually have our own staff across all of the shifts in a week, days and nights, we just don’t have enough people and I think the mums do notice when we’ve had a lot of bank staff … that aren’t very familiar with the ward.” (MBU, senior manager)*

One MBU clinician did, however, report feeling that MBUs, due to being specialist units, attract staff who are particularly passionate about perinatal care and felt this was reflected in staff’s commitment to their jobs.

*“I think MBUs attract certain types of staff because you’re passionate about this specific area … I think if you end up working here it’s because you really like it … I think it’s just we are more motivated coming to work.” (MBU, nurse)*

##### Therapeutic environment

On MBUs, the formation of positive relationships was also perceived to be facilitated by their more homely, comfortable and modern environment. Participants attributed this to their décor and facilities, including home comforts and children’s toys. This helped make them more welcoming, family-friendly environments. Women who had experience of admissions to both settings often commented on the more comfortable MBU environment.

*“Compared with the adult acute wards you know, it’s all crafted and it’s kept really nice and the area where you can make tea and coffee is kind of, it feels like a, just a nice dining room and everything’s clean and they’ve got nice mugs from Waitrose [supermarket] and just things, small things like that which make a whole lot of difference.” Mother 8*

In contrast, general psychiatric wards were typically more clinical, impersonal and dated environments. Some women likened them to prisons or old-style psychiatric hospitals: frightening environments not conducive to recovery.

*“It wasn’t just an old building, it was old facilities, it was just so clinical and dated. You felt like you were in some kind of psychiatric mental movie from the 1950s … that isn’t an environment to get better in. It just depressed me even more.” Mother 4*

While women on MBUs also had their own private rooms, which they viewed as safe havens where they could retreat, mothers on an acute psychiatric wards at times described a lack of appropriate facilities for postpartum women and/or having to share spaces with others who were not understanding of their needs as new mothers.

*“The toilet was horrible, the shower was horrible … And obviously post birth you need … clean sanitation and like, so I was bleeding a lot still … I needed a bath, but you don’t have baths in your room.” Mother 14*

*“That’s one of the things about being with other people in a room, because others who were mothers would have understood when they were hearing me express, but the lady who was next door to me … just hearing the sound of it was getting to her. So I used to … take my pot machines and sometimes it would be at a stupid time of the night and I would have to go to the bathroom and pump in there.” Mother 13*

These comparisons were also consistent with a lack of freedom, choice, control and power more commonly described on general psychiatric wards. However, across both MBUs and general wards, some women reported wanting greater involvement in decisions about their care, finding it hard to adjust to all the rules, and disliking procedures such as bag and room searches, and supervised family visits, experiencing these as intrusive and disempowering.

*“It was hard at first [on the MBU] because they had all these rules and you couldn’t, you know, bath your baby on your own and you couldn’t change your baby on your own. You had to be supervised to do most things. So that was just kind of like, you felt like you were imprisoned sometimes.” Mother 1*

While clinicians could understand women’s dissatisfaction with these aspects of their care, from their point of view strict rules and regulations were seen as very important, with activities like co-sleeping considered to present unacceptable risks.

*“I think [the rules] don’t always work well for the mums because when they go home they won’t be doing what we expect them to do here. But we’re in a psychiatric hospital with very unwell women and other babies so we have to think about safety first. So things about co-sleeping, not walking around holding your baby, not having your baby in a buggy on a strap, all of those things, we have to have those rules here.” (MBU, senior manager)*

#### Access and post-discharge experiences

##### Barriers to access

Although MBUs were preferred to general wards, lack of beds and long home-MBU distances could present significant barriers to access, with some women opting for admission to a local general psychiatric ward as a result. Many women voiced the need for increased MBU provision.

Clinicians noted that long home-MBU distances could be a challenge for professionals too. For example, it made it time-consuming for MBU staff to visit women at home, and for community teams to visit women at the MBU. Efforts were made to share travelling responsibility between teams to reduce this burden, though it was still challenging for staff.

*“Sometimes when they’re very far I feel sometimes the community teams do make heroic efforts to come to the MBU and have an opportunity to have a face-to-face meeting. Or we might try and facilitate by having the final discharge meeting in the community and our staff travelling there. But that is a real challenge for everyone I think.” (MBU, psychologist)*

Though several women experienced delays accessing MBUs, some were able to access them quickly. In these cases, women attributed their ease of access to adequate preparation. This involved women and professionals being proactive, with one woman even prospectively reserving a bed in anticipation of mental health difficulties post-birth.

Clinicians spoke about their own difficulties dealing with uncertainty around timescales for admissions to MBUs, for example where staff were not sure when a bed would become available, or when plans changed because a higher priority case arose. Clinicians reported that this could be frustrating for everyone.

*“I think one of my most frustrating jobs is … managing all the referrals … we have a lady, we see the referral, yes she’s definitely appropriate, she’s on an acute ward … And then we get an emergency referral, this one’s at home, it’s very risky, we have to prioritise that referral. So sometimes if you’re on an acute bed you actually get pushed further down the list because you’re already in a safe place … we can never really give an accurate description as to when someone can come in because the picture is a very fluid” (MBU, nurse)*

General ward clinicians said this uncertainty could result in professionals, women and their families alike becoming pre-occupied about arranging potential transfers, detracting focus from supporting women as best as possible whilst on the general ward.

*“That was the focus, when’s she going to go, when's she going to go, she’ll go today? She might go today. Family, ‘is she going today, why isn’t she going today?’ … and actually if she was happy here, which I think she kind of was, and she was getting to see her child, and we were facilitating that … did we need to focus so much on this transfer?” (Acute ward, foundation doctor)*

MBU clinicians also reported receiving inappropriate referrals from generic mental health services including general wards, which they attributed to a lack of perinatal expertise, including a lack of clarity around MBU referral criteria and the model of care on MBUs. MBU staff felt that more out-reach training by MBU staff – not just to general wards but community teams as well – could help, as well as having community staff spend a day on an MBU and vice-versa. However, they also felt that pressure to free up beds on general wards sometimes resulted in staff referring women to MBUs who were too high risk. MBU staff said they had started conducting in-reach assessments on general wards in an attempt to prevent inappropriate transfers.

MBU staff suggested that the introduction of high-dependency MBUs, or high-dependency sections within existing MBUs, could be valuable. This would help MBUs cater for the needs of higher-risk cases, who are currently admitted to general wards and separated from their babies.

*“There are no perinatal high dependency areas in the country. If we had a little self-contained area where we could nurse maybe two women who were really unwell, with increased staffing numbers, that they would get the perinatal expertise that they need, but also they would be safe and everyone else would be safe. So I think that’s something else that the government need to look at.” (MBU, senior manager)*

##### Poor continuity of care post-discharge

As well as difficulties accessing MBUs, most women reported a lack of accessible psychological, practical, childcare and social support following discharge from both MBUs and general psychiatric wards. This poor post-discharge continuity of care contributed to challenging hospital-to-home transitions in which mothers felt isolated and lacked confidence in their ability to cope. Many experienced adjustment difficulties upon returning home, finding it difficult to integrate back into family life and reassume childcare responsibilities in the absence of external support.

*“What was the point of going to [an MBU] if it doesn’t get followed up at all? I still don’t understand.” Mother 3*

Poor communication between services, long waiting lists, and not having a consistent point of contact were cited as obstacles to accessing post-discharge support. This, combined with inadequate information provision to mothers about support available, meant that mothers often had to rely on informal support from family members once home. Whilst phased discharges in both general psychiatric wards and MBUs did help women feel more prepared for life at home, it could be emotionally difficult for women’s older children.

*“I started to have trips home and I found that those were quite difficult with my son because he, when I came home he would want to know how long I was staying and he was finding it really distressing that I would be home for a couple of days and then suddenly I was gone again.” Mother 8*

Clinicians from both service contexts recognised that transitions home following admission can be challenging for women, perhaps particularly so after longer admissions and after receiving high-quality, specialist support in an MBU. They felt this could sometimes foster dependency on services.

*“We do end up running ourselves quite ragged and doing everything for somebody who actually is able … rather than empowering someone to do stuff for themselves” (MBU, nurse)*

*“I think we can be quite womb-like and I think we really cosset and nurture and take care of the mums. Although I think that’s absolutely what they need, I think that can be quite hard then when they’re being discharged because they really struggle to move on from the care and the support and the nurturing that they’ve had here, sometimes for the first time in their life.” (MBU, senior manager)*

Like women, clinicians felt gradual discharges could help women manage the transition home, as well as closer liaison with community teams to aid discharge-planning. They also said expanded provision of specialist perinatal support in the community could help, particularly around infant-care.

*“Quite recently as well the perinatal community teams have expanded as well to include nursery nurses as well. So I think that might bridge a little bit of a gap. But I do think it would also be helpful if the team here communicated with the community teams more just to transition a bit more smoothly … These are staff who don’t really know these mums so perhaps we could work together a bit.” (MBU, nursery nurse)*

Clinicians noted that perinatal expertise and resources within community teams is dependent on location, with some areas having specialist perinatal community teams, and others having none. Generic mental health teams (e.g. crisis teams and community teams) were reported to generally lack perinatal expertise, and to operate in a way that was not always well-suited to the needs of new mothers (e.g. inconvenient visiting schedules). MBU staff noted that some generic community teams have ‘perinatal champions’ – dedicated members of staff with perinatal expertise who perinatal women should be allocated to, though these procedures were reported to not always be correctly followed in practice.

Staff felt that it could be valuable for there to be a specialist perinatal equivalent of crisis teams (which can serve as an intermediate team between inpatient and community services). However, staff from both service contexts said that, in the absence of this, more training and consultation by perinatal services would be valuable to improve perinatal care provision within generic mental health teams.

*“Probably closer link working between community teams and home treatment teams as well, or MBUs and home treatment teams so we can offer our expertise to get to them.” (MBU, nurse)*

## Discussion

This study supports previous research indicating an overall preference for MBUs over general psychiatric wards amongst women experiencing severe perinatal mental health problems and requiring inpatient care. Women and clinicians alike saw value in the more specialist, perinatally-focused care provided on MBUs over the more generic care on general psychiatric wards, in-line with previous research indicating a preference amongst women and professionals for more specialist perinatal mental health care [27]. Also consistent with previous research, most women preferred not to be separated from their babies [10, 11], viewing co-admission as an important facilitator of their recovery, and valuing the parenting and practical childcare support they received on MBUs. They felt this specialist parenting support promoted positive mother-baby relationships, and improved their confidence in their parenting abilities. This supports previous research suggesting that co-admission to a MBU can lead to improvements in maternal well-being and mother and baby relationships on discharge [28, 29] and may protect against detrimental effects of maternal mental health problems and separation on children’s long-term developmental outcomes [12, 30]. It could be hypothesised that the parenting support and interventions received during co-admission to an MBU could help to foster more positive mother-baby attachments [31] – perhaps by improving maternal sensitivity, a key theoretical aspect of parenting related to attachment [32, 33]. More research is needed to explore this, since national guidance on parenting interventions in MBUs is currently limited [34, 35], due to a lack of evidence for the effectiveness of parenting interventions for women experiencing severe mental illness, especially in MBU settings [36].

Participants viewed positive relationships with staff as of central importance on both general psychiatric wards and MBUs. This was also a key finding of a qualitative meta-synthesis of research examining service users’ wider experiences of psychiatric inpatient care [15]. In common with the wider mental health literature, women valued staff who appeared committed to their jobs and were empathetic, compassionate and warm towards them [17, 18]. Some participants reported negative interactions with staff who lacked these personal qualities and dedication to their job. Limited staff availability, due to heavy workloads and understaffing, and associated lack of staff continuity were identified by both women and clinicians as barriers to therapeutic alliances with staff, as in previous research in other acute inpatient settings (including crisis houses) [18] and crisis resolution and home treatment teams [37].

Many women also valued informal peer support from other women experiencing perinatal mental health problems in MBUs. This helped normalise women’s difficulties and fostered a sense of ‘community’. This complements a wider research literature indicating that more formal peer support (e.g. self-help style services, befriending and peer support workers) may help promote recovery from perinatal mental health difficulties, and mental health difficulties more broadly, both in inpatient environments and in the community [38–40]. Participants typically reported fewer positive relationships with other patients on general psychiatric wards. Many felt less understood, and disturbed by other services users’ more diverse and severe presentations. The typically less ‘home-like’ environment in general psychiatric wards may also have hindered bonding with peers [41].

Despite the clear preference for MBUs, women and clinicians alike described difficulties accessing MBUs, especially due to unavailability of beds and long distances between home and MBUs, in-line with previous research [27, 42]. Accessibility issues could cause anxiety and stress for women and clinicians alike. Indeed, access difficulties have been widely-reported across the perinatal mental health care pathway and across mental health services more generally in England, attributed in part to inadequate funding, lack of integration of services, insufficient training and an understaffed workforce [4, 43]. However, as already mentioned, since the study period there has been a significant increase in perinatal mental health service provision following an increase in funding for four new mother and baby units in regions underserved, and the expansion of specialist community perinatal mental health services.

Some women on both MBUs and acute wards also experienced considerable difficulties transitioning back home, and clinicians recognised a need to support women more with this. This complements previous research calling for more collaborative and integrated services in the perinatal period [43–45], with evidence suggesting that this may improve both perinatal and infant mental health outcomes [44, 46].

### Clinical and policy implications

This study suggests that specialist inpatient care is considered preferable to generic care in the perinatal period from both service user and staff perspectives, and reveals some of the problems that insufficient provision of specialist perinatal services can cause. The findings support recent increased investment to develop more MBUs in the UK and suggest that there may be value in introducing this model of care to countries that do not currently have specialist facilities. Clinicians also suggested there may be value in developing specialist perinatal community crisis teams and high-dependency MBUs, to cater for a wider population of perinatal women. International solutions to the problem of limited MBU funding could also include: only accommodating babies during the day-time [47] and only operating MBUs on weekdays [9]. Furthermore, access to alternative services (such as mother-child day-care units, community-based and home-based services) may help to compensate for limited MBU access in some areas [9]. Further research comparing the benefits, limitations, feasibility and cost-effectiveness of such models of care is needed, including international models across high, middle and low resource countries.

The lack of perinatal expertise and focus on general psychiatric wards was a clear barrier to delivering high-quality, person-centred care tailored to perinatal women’s needs. Clinicians suggested that this could be addressed by more perinatal mental health training, closer liaison between non-perinatal and perinatal services, and greater focus on adaptations to the ward environment to better cater for women’s needs. Even on MBUs, the findings suggested a need for better facilities to support women whose babies are older, as well as more support with specific aspects of infant-care, and greater efforts to ensure women do not feel disempowered by strict MBU rules.

This study also revealed a need for better post-discharge support for women. Transitioning from inpatient care to home living can be a difficult time for any psychiatric inpatient [15]. However, this study’s findings demonstrate that women admitted for perinatal mental health difficulties face additional challenges during this transitionary period. For instance, many reported difficulties reintegrating back into their family unit, adjusting to family life and providing childcare in the absence of external support. Many women lacked confidence in their ability to cope at home, even - and perhaps especially - after receiving intensive parenting support in an MBU. This highlights the importance of connecting mothers with mental health and parenting services following discharge [48, 49]. Such support could help prevent relapse and improve mothers’ functioning in the community [50, 51]. Clinicians also emphasised the need for closer collaboration between inpatient services and relevant community-based and home-based services and organisations.

Finally, more efforts also need to be made by both acute wards and MBUs to cater for women’s families. This is imperative given the importance of social support for women experiencing perinatal mental health problems [40]. Support participants found helpful that could be more widely implemented included: reimbursement of travel expenses; flexible visiting hours; and provision of a safe, homely, child-friendly environment (for example, by providing children’s toys and access to a private family room for visits). In some countries, such as France and Australia, some MBUs even permit fathers to reside in them alongside the mother and baby [9].

### Research implications

This study builds on existing literature and highlights a need for future research investigating: differences in MBU and general ward outcomes in quantitative terms; which models of care work for whom (and factors mediating this); and whether beneficial features of MBUs could be implemented on general wards to improve perinatal care provision in this service context. Future studies could also explore international differences in MBU operation and outcomes, and evaluate other alternative models of specialist perinatal inpatient care. Such research would provide further insights into how best to support women experiencing severe perinatal mental health problems, and so could further inform clinical practice, service commissioning and policy-making internationally.

### Strengths and limitations

For this study, participants were successfully recruited from nine NHS healthcare providers across England, and the purposive sampling strategy ensured a diverse sample of women who varied in their demographic and clinical characteristics. Nevertheless, it is possible that recruitment of women via clinicians may have led to hearing the views of a more engaged or satisfied sample and it was notable that the sample was overall fairly highly educated.

Similarly, as the practitioner sample was drawn from only one MBU and one acute ward - and specifically a female ward rather than a mixed ward - the findings may not apply to all MBU or acute ward teams. It is also possible that the focus group methodology could have restricted the information obtained, particularly as each group included colleagues of different levels of seniority. Nevertheless, the fact that both women’s and clinicians’ views were accessed is a strength of the current study, plus their similarities arguably increase confidence in the findings.

The fact that most interviews were conducted by a female clinical psychologist with considerable perinatal experience may have facilitated open discussion, but could also have inhibited it by creating a power imbalance between the women and interviewer, especially in cases where participants had previous negative experience with healthcare professionals.

Finally, as this study was part of a wider project on women’s entire pathway of care during the perinatal period, the length of time in interviews spent specifically discussing MBUs or acute wards was sometimes limited. Nevertheless, rich information was obtained, allowing identification of many relevant themes.

## Conclusions

This qualitative study compared experiences of being admitted to general psychiatric wards versus MBUs for acute, severe perinatal mental health difficulties. It revealed an overall preference for specialist MBUs over general psychiatric wards, with most women valuing being able to be co-admitted with their baby to an MBU. Women and clinicians alike described MBUs as generally more child-friendly environments, offering greater perinatal expertise and a more inclusive, family-centred approach to care. MBUs were felt to be better-suited to the needs of new mothers. Future research should focus on how these insights can be used to deliver better-quality person-centred care on general psychiatric wards.

The findings support the current drive for increased investment into MBU service provision in the UK to address the significant problems caused by their currently limited accessibility. Further research investigating the cost-effectiveness of MBUs compared to alternative models of care both within the UK and internationally is also needed to inform future policy-making and commissioning of mental health services.

Key areas of dissatisfaction common to both service contexts included: lack of psychological and practical support for partners and relatives, and poor post-discharge continuity of care. Participants’ experiences were also mixed with regard to factors such as staff availability and therapeutic relationships with staff. These are commonly-recurring themes across the psychiatric inpatient literature. Suggestions were made for how to address these concerns and improve the quality of inpatient care provided to women experiencing perinatal mental health problems. However, further qualitative research exploring the views of partners/relatives, staff and commissioners is also needed.

## Data Availability

The datasets generated and/or analysed during the current study are not publicly available due to them containing information that could compromise research participant privacy/consent but are available from the corresponding author on reasonable request.

## Abbreviations

MBU: Mother and Baby Unit
PAG: Perinatal Service User and Carer Advisory Group
